# Synthesis, Docking Study and β-Adrenoceptor Activity of Some New Oxime Ether Derivatives

**DOI:** 10.3390/molecules19033417

**Published:** 2014-03-20

**Authors:** Hazem A. Ghabbour, Eman R. El-Bendary, Mahmoud B. El-Ashmawy, Mohamed M. El-Kerdawy

**Affiliations:** 1 Department of Medicinal Chemistry, Faculty of Pharmacy, Mansoura University, Mansoura 35516, Egypt; 2 Department of Pharmaceutical Chemistry, Faculty of Pharmacy, King Saud University, P. O. Box 2457, Riyadh 11451, Saudi Arabia

**Keywords:** synthesis, screening, docking, oxime ethers, adrenergic, β-receptors

## Abstract

A new series of oxime ethers **4a**–**z** was designed and synthesized to test the blocking activity against β_1_ and β_2_-adrenergic receptors. Docking of these ether derivatives into the active site of the identified 3D structures of β_1_ and β_2_-adrenergic receptors showed MolDock scores comparable to those of reference compounds. Biological results revealed that the inhibition effects on the heart rate and contractility are less than those of propranolol. Nevertheless, the two compounds **4p** and **4q** that displayed the highest negative MolDock score with β_2_-adrenergic receptors showed β_2_-antagonistic activity by decreasing salbutamol relaxation of precontracted tracheal strips, which indicates the importance of a chlorothiophene moiety in the hydrophobic region for best complementarity with β_2_ receptors. On other hand, the presence of a homoveratryl moiety increases the MolDock score of the tested compounds with the β_1_ receptor.

## 1. Introduction

**Figure 1:**
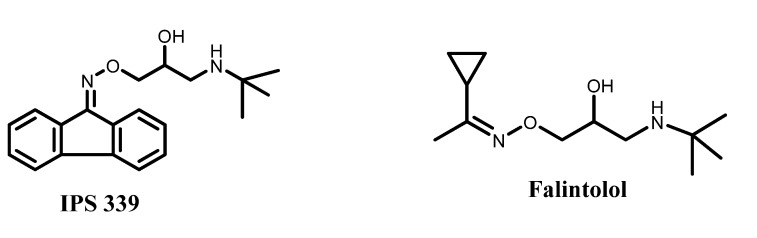
Chemical structures of IPS 339 and falintolol.

β-Adrenoceptor blockers represent an important class of therapeutics due to their usefulness in a number of diseases, such as angina pectoris, arrhythmia and hypertension. They may also be used in migraine, glaucoma, tremors, hyperthyroidism, and anxiety-provoking situations [1]. Based on the chemical structure of the classical aryloxypropanolamine β-adrenoceptor blockers, Imbs *et al.* [2] showed that the insertion of an oxime ether in place of the typical ether linkage retained β_2_-antagonist activity; e.g., IPS 339 (Figure 1). Other oxime ethers were also synthesized and reported to have β_2_-blocking activity with high potency, e.g., falintolol (Figure 1) [3,4]. Although the above mentioned cardiovascular effects are mainly attributed to β_1_-blockage, β_2_-adrenoceptor antagonists might be potentially interesting as experimental tools [5], or for testing in pulmonary arterial hypertension [6].

In the present investigation, we intended to further explore the general structure of oxime ethers through different molecular modifications, and to test the β_1_- and β_2_-adrenoceptor blocking activity of the new compounds. The manipulation strategy involves usage of aromatic, alicyclic, bicyclic and/or heterocyclic moieties to represent the hydrophobic part of the molecule, in order to verify the importance of these moieties in binding with the receptor, and hence on the selectivity of the resulted compounds. Obeying the SAR of the β-blockers, the already known β-directing isopropyl and *tert*-butyl group as well as the dimethoxyphenylethyl moiety were selected as the best representatives of alkyl and arylalkylamines. The synthesis, molecular docking and preliminary biological evaluation of the thus designed *O*-(3-alkylamino-2-hydroxypropyl)oxime derivatives **4a–z** are reported herein.

## 2. Results and Discussion

### 2.1. Chemistry

**Scheme 1:**
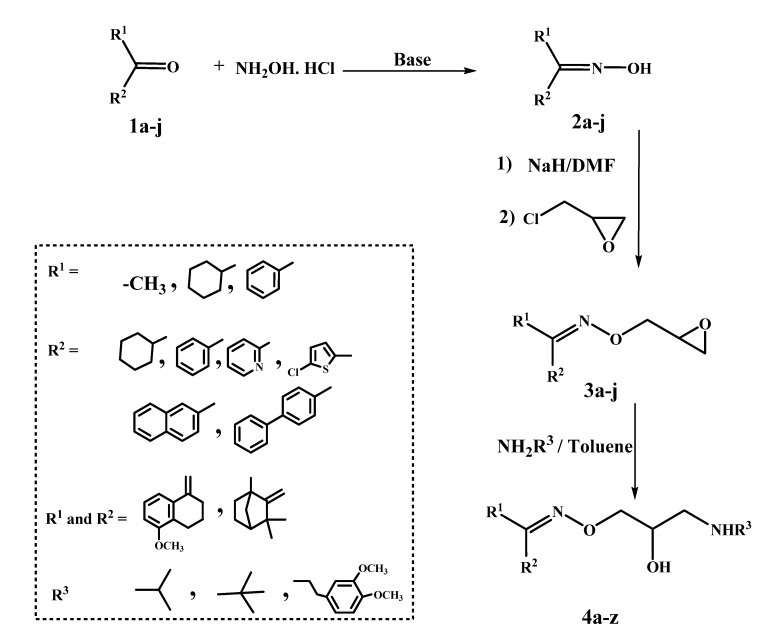
Synthesis of the oxime ethers **4a**–**z**.

The general method to prepare the final compounds **4a–z** is outlined in Scheme 1. The oximes **2a**–**j** (Table 1), required in the present work, have been obtained via reaction of the corresponding ketones **1a**–**j** with hydroxylamine hydrochloride, as previously reported [3,7,8,9,10,11,12,13,14]. The two oxime geometric isomers were not separated, based on the previously reported absence of appreciable difference in β- blocking activity between the *E-* and *Z-*isomers of the final oxime ether derivatives [15]. Accordingly, compounds **2a**–**j** were reacted with epichlorohydrin, in dry DMF, in the presence of NaH, to afford the epoxy compounds **3a**–**j** (Table 1). In the ^1^H-NMR spectra, the oxirane protons appeared mostly as multiplets at δ 2.4–3.0 ppm, while the -OCH2- protons appeared as two doublets of doublets at δ 3.7–4.4 ppm (Experimental part). The target compounds **4a–z** (Table 2) were prepared by reaction of the epoxy compounds **3a**–**j** with excess of isopropylamine, *tert*-butylamine or homoveratrylamine in toluene, in a sealed tube, at 120 °C.

**Table 1:** Oximes **2a**–**j** and oxime ethers **3a**–**j**. 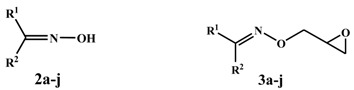

Compound No.	R^1^	R^2^	Compound No.	R^1^	R^2^
**a**	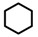	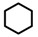	**f**	CH_3_	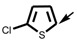
**b**	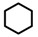	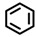	**g**	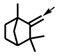	
**c**	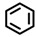	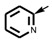	**h**	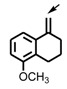	
**d**	CH_3_	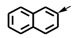	**i**	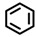	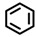
**e**	CH_3_	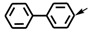	**j**	CH_3_	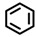

### 2.2. Molecular Docking

Molecular docking is a very popular method employed to investigate molecular association. It is particularly useful in the drug discovery arena to study the binding of small molecules (ligands) to macromolecules (receptors or enzymes). Docking-based drug design using structural biology remains one of the most logical and esthetically pleasing approaches in the drug discovery process. The structured knowledge of the binding capabilities of the active site residues to specific groups on the agonist or antagonist leads to various proposals for the synthesis of very specific agents with a high probability of biological action [16].

**Table 2:** *O*-(3-Alkylamino-2-hydroxypropyl)oxime derivatives **4a**–**z**. 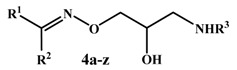

Compound No.	R^1^	R^2^	R^3^	Compound No.	R^1^	R^2^	R^3^
**4a**	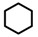	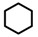	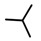	**4n**	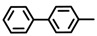	CH_3_	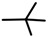
**4b**	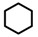	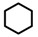	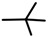	**4o**	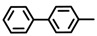	CH_3_	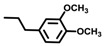
**4c**	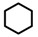	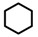	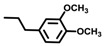	**4p**	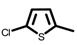	CH_3_	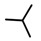
**4d**	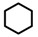	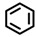	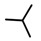	**4q**	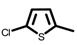	CH_3_	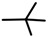
**4e**	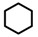	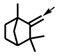	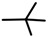	**4r**	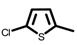	CH_3_	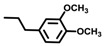
**4f**	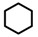	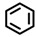	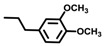	**4s**	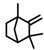		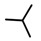
**4g**	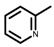	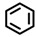	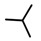	**4t**	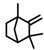		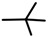
**4h**	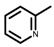	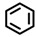	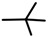	**4u**	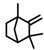		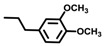
**4i**	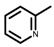	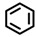	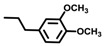	**4v**	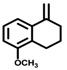		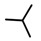
**4j**	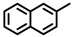	CH_3_	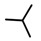	**4w**	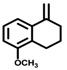		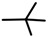
**4k**	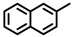	CH_3_	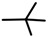	**4x**	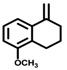		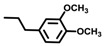
**4l**	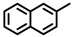	CH_3_	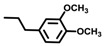	**4y**	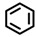	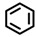	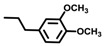
**4m**	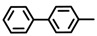	CH_3_	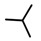	**4z**	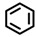	CH_3_	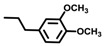

**Figure 2:**
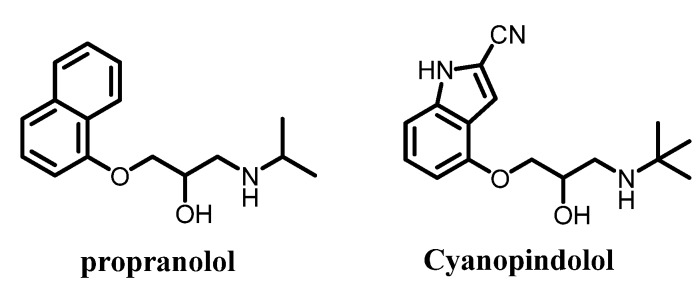
Chemical structures of propranolol and cyanopindolol.

Twenty six new oxime ethers **4a**–**z** were subjected to the docking study into the identified β_1_ and β_2_- adrenergic receptor active sites [17,18,19]. The docking technique using the Molegro Virtual Docker [20] is used to estimate the binding affinity between external ligands and structurally-known biological macromolecules, and hence, to predict the biological importance of these ligands [19,21]. The forces of interaction were declared and scoring functions were determined. Propranolol (a non-selective β-antagonist), cyanopindolol (selective β1-antagonist) (Figure 2) and IPS 339 (selective β2-antagonist) were used in this docking study as reference molecules. Snapshots for all prepared compounds were taken to reveal their molecular interactions (hydrogen, hydrophobic and/or ionic bonds) with the amino acids of β_1_ and β_2_- receptor active sites. MolDock scores and the energy H-bond interactions between these compounds and receptor were calculated (Table 3). The results showed that all tested compounds have affinity for β1and β2-adrenoceptors comparable to that of the reference compounds.

**Table 3:** MolDock scores and hydrogen bond energy for compounds **4a**–**z** and reference compounds with the β_1_ and β_2_ receptors.

Comp.	β_1_-Receptor		β_2_-Receptor	
	MolDock Score	H-Bond E.	MolDock Score	H-Bond E.
**4a**	−115.13	−2.5	−100.806	−4.35757
**4b**	−122.697	−4.58252	−99.6442	−2.42254
**4c**	−142.285	−5.19707	−105.6596	−3.42955
**4d**	−121.41	−3.59754	−86.4381	−1.16172
**4e**	−127.903	−3.96668	−88.3577	−0.04374
**4f**	−153.964	−5	−95.1594	0
**4g**	−125.653	−2.5	−92.7053	−3.4013
**4h**	−124.044	−2.83065	−85.3741	−0.69094
**4i**	−145.546	−5.2648	−90.616	−2.98878
**4j**	−114.594	−2.62113	−79.1286	0
**4k**	−120.406	−2.5	−90.5964	−2.63215
**4l**	−155.836	−6.1153	−103.123	−0.16123
**4m**	−125.091	−3.98536	−74.7409	−0.97756
**4n**	−120.574	−2.18542	−73.6041	−3.78389
**4o**	−167.406	−4.49787	−91.127	−2.5557
**4p**	−105.452	−1.63375	−113.5079	−4.03237
**4q**	−114.132	−2.62848	−117.075	−4.34578
**4r**	−143.244	−2.15101	−102.028	−0.41247
**4s**	−108.994	−1.50057	−85.4672	−3.26981
**4t**	−108.574	−2.79887	−84.921	−0.78187
**4u**	−148.747	−4.41468	−100.887	−0.03188
**4v**	−105.443	−1.00129	−84.9409	−1.98422
**4w**	−105.034	0	−85.1195	−0.99963
**4x**	−139.094	−4.07406	−99.449	−0.47872
**4y**	−152.107	−5.54788	−93.6354	−2.4702
**4z**	−125.4	−2.28939	−84.4559	−2.16834
**Propranolol**	−110.517	−2.53142	−88.1631	−3.92249
**IPS 339**	−104.212	−2.91744	−95.141	−2.87182
**Cyanopindolol**	−129.204	−10.7754	−85.6768	−2.98876

**Figure 3:**
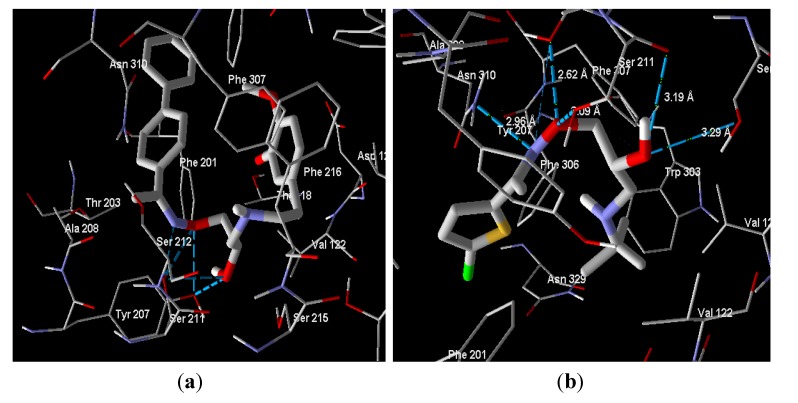
(**a**) Docking snapshot of compound **4o** with β_1_ receptor. (**b**) Docking snapshot of compound **4q** with β_2_ receptor. Compounds are represented as stick (thick lines) while the amino acids of the active site of the β2-adrenoreceptor appear as (light) lines. Hydrogen atoms in both ligands and receptors have been omitted for clarity. Blue dashed lines indicate the hydrogen bonds.

In the β_1_-receptor, the selective antagonist cyanopindolol has a MolDock score −129.2 with H-bond energy −10.7 and compounds **4c**, **4f**, **4i**, **4l**, **4o**, **4r**, **4u**, **4x** and **4y** have better MolDock scores than cyanopindolol. The best compound in this study was **4o**, which has MolDock score of −167.4 and forms five hydrogen bonds with the amino acids Ser 211 and Tyr 207 in the active site of the receptor Moreover, hydrophobic interactions probably existed between compound 4o and Phe 201, Phe 216 and Phe 307 (Figure 3a). It is worth mentioning here that all tested compounds which have better MolDock score than the reference drug have in its structure the homoveratryl moiety which may increase the quality of binding between the ligands and receptor.

In the β_2_-receptor, only six compounds **4a**, **4b**, **4c**, **4l**, **4u** and **4x** have slightly lower MolDock scores than the β_2_-antagonist IPS 339, while compounds **4p** and **4q** displayed the highest negative MolDock scores that indicated highest complementarity with β_2_-adrenoceptors (Table 3).

Figure 3b represents the snapshot for compound **4q** and illustrates the different amino acids of the binding site that are involved in the compound-receptor interaction, where more hydrogen bonds and hydrophobic interactions were involved for this compound than others. Hydrogen bonding interactions were β-hydroxyl group in **4q** with the peptide carbonyl group of Ser 215 (3.29 Ǻ) and with the free hydroxyl group of Ser 311 (3.19 Ǻ), oxygen of the oxime moiety makes a hydrogen bond with Ser 211 and Ser 212 (3.09 and 2.62 Ǻ, respectively) and the nitrogen of the oxime moiety also interacts with Asn 310 by hydrogen bonding (2.96 Ǻ). Hydrophobic interactions may be found between the thiophene ring and the amino acids Phe 201 and Phe 306. 

### 2.3. Biological Evaluation

Blocking of the β_1_-adrenoceptor prevents the increase in the heart rate and contractility caused by epinephrine, while β_2_-adrenoceptor antagonism results in bronchospasms, uterine contraction and blood vessel spasms [22]. In the present investigation, certain newly synthesized compounds were subjected to *in vitro* biological testing for their blocking activity against β_1_ and β_2_-adrenoceptors, using isolated guinea pig atria and trachea, respectively, according to reported procedures [23,24,25]. Meanwhile, the agonistic activities at both receptors were also recorded.

#### 2.3.1. *In Vitro* Screening for β_1_-Adrenoceptor Activity in Isolated Guinea Pig Atria

Seventeen of the newly synthesized compounds were subjected to an *in vitro* biological screening for β_1_-adrenoceptor activities using isolated atria of guinea pigs. The available results indicate that six of these compounds (**4f**, **4i**, **4l**, **4r**, **4y** and **4z**), with 3,4-dimethoxyphenethyl groups at the terminal amine produced 22%–31% inhibition in the heart rate and 30%–55% inhibition in the contractility of the atria. Propranolol, the reference drug, produced 70 and 60% inhibition of the heart rate and contractility, respectively (Table 4).

**Table 4:** Thepercentinhibitionintheheartrateandthecontractilityoftheisolatedguineapigatriacausedby the tested compounds.

Compound No.	β1-antagonist		Compd. No.	β1-antagonist	
	% inhibition in heart rate	% inhibition in contractility		% inhibition in heart rate	% Inhibition in contractility
**4a**	3%	0%	**4o**	1%	0%
**4b**	2%	0%	**4p**	6%	0%
**4c**	6%	1%	**4q**	2%	0%
**4e**	0%	0%	**4r**	31%	55%
**4f**	29%	30%	**4u**	2%	0%
**4g**	2%	0%	**4x**	0%	0%
**4i**	25%	40%	**4y**	26%	35%
**4k**	3%	1%	**4z**	22%	30%
**4l**	28%	45%	**Propranolol**	70%	60%

Replacement of the 3,4-dimethoxyphenethyl group with isopropyl or *tert*-butyl groups showed loss of β_1_-adrenergic antagonist activity, as in **4g**, **4e**, **4k** and **4p**. It is worth mentioning that the dimethoxy substitution on the phenethylamine was reported to be the preferable aralkylamine moiety required to achieve β-adrenergic receptor blocking activity [26]. On the other hand, compounds **4c**, **4o**, **4u** and **4x** contain 3,4-dimethoxyphenethylamine groups, but they did not show any β_1_-adrenergic antagonistic activity. This might be explained by the structural difference on the other side of the oxime moiety, where the hydrophobic part might be improperly oriented to fit in the active site in the β_1_-adrenergic receptor. Meanwhile, the study also indicated that none of the tested compounds has β_1_-agonistic activity; whereby no increase in the heart rate or contractility of isolated guinea pig atria was noticed.

#### 2.3.2. *In Vitro* Screening for β2-Adrenoceptor Activity in Isolated Guinea pig Trachea

Eleven of the newly synthesized compounds were subjected to an *in vitro* biological screening for β_2_-adrenoceptor activities using isolated trachea of guinea pigs. The test depends principally on the relaxation effect of salbutamol on the pre-contracted tracheal muscle induced by acetylcholine. Compounds of potential antagonistic activity are expected to decrease the relaxation caused by salbutamol. The available results indicated that only two compounds, **4p** and **4q**, decreased the salbutamol relaxation of pre-contracted tracheal strips, while the rest of the tested compounds did not show any β_2_-antagonist activity. The results also showed the absence of agonistic activity for all tested compounds (Table 5).

**Table 5:** β_2_-Adrenergicactivitiesof thetested compounds.

Compound No.	β2-agonist	β2-antagonist	Compound No.	β2-agonist	β2-antagonist
**4a**	-	-	**4p**	-	+
**4e**	-	-	**4q**	-	+
**4g**	-	-	**4r**	-	-
**4i**	-	-	**4t**	-	-
**4k**	-	-	**4v**	-	-
**4m**	-	-	**Propranolol**	-	+

(+) decreased relaxation, (−) no effect.

Taken together, docking of twenty six compounds into the β_1_ and β_2_ adrenergic receptors has been studied, and the energy of interaction has been calculated. The energy of interaction revealed that compounds **4p** and **4q** indicated the highest complementarity with β_2_-adrenoceptors (Table 3). These two compounds, in particular, decreased the salbutamol relaxation effect on guinea pig trachea, suggesting their β_2_-antagonist activity. The structural characteristics that might differentiate compounds **4p** and **4q** from others is the presence of a chlorothiophene moiety in the hydrophobic region. Compounds with chlorothienyloxypropanolamine moieties were previously studied by El-Ashmawy *et al.* [27] for their β-blocking and partial agonist activity, but no tests were done with β_2_-receptors. Compound **4r** also has the same hydrophobic chlorothienyl moiety, however, the presence of a bulky homoveratrylamine portion might explain its inactivity at the β_2_-adrenoceptors. It is worth mentioning that this compound (**4r**) displayed the highest percentage inhibition on β1-adrenoceptor (55% inhibition of the contractility) (Table 4). These findings confirm the significance of a homoveratrylamine portion for β_1_ receptors, especially in view of the data from the β_1_-adrenergic receptor docking study (Table 4); and indicate the importance of the presence of a chlorothiophene moiety in the hydrophobic region for best complementarity with β_2_ receptors.

## 3. Experimental

### 3.1. General Information

Melting points (°C) were recorded using a Fisher-Johns melting point apparatus and are uncorrected. ^1^H-NMR spectra were obtained on FT-NMR spectrometer (200 MHz) Gemini Varian using TMS as internal standard (chemical shifts in ppm, δ units). MS analyses were performed on JEOL JMS-600H spectrometer. All the ketones used were commercially available from Sigma-Aldrich Chemical Company (Munich, Germany), except benzophenone and acetophenone that were purchased from El-Nasr Pharmaceutical Chemicals Company, Cairo, Egypt. Oximes **2a**–**j** were prepared following the procedures reported in the literature [3,7,8,9,10,11,12,13,14]. Two of the oxirane compounds **3i** and **3j** were previously reported in the literature [14].

#### 3.1.1. General Procedure for Synthesis of *O*-Oxiran-2-ylmethyl Oximes **3a**–**h**

A mixture of the appropriate oxime **2a**-**h** (0.01 mol) and NaH (0.26 g, 0.011 mol) was stirred in dry DMF (30 mL) at room temperature for 10 min. A solution of epichlorohydrin (1.1 g, 0.01 mol) in dry DMF (5 mL) was added dropwise over a period of 10 min. The resulting mixture was further stirred for 36 h at room temperature (TLC). The reaction mixture was poured in ice water (200 mL) and extracted with chloroform (3 × 100 mL). The organic layers were collected, washed twice with brine solution, dried over anhydrous MgSO_4_, and the chloroform was evaporated under reduced pressure to obtain the desired compounds in pure form.

*Dicyclohexylmethanone O-oxiran-2-yl methyl oxime* (**3a**). Yield 55%; M.p. 120–122 °C; ^1^H-NMR (CDCl_3_) δ 1.15–1.70 (m, 20H, 10CH_2_ in cyclohexane), 2.21–2.44 (m, 2H, 2CHC=NO), 2.54–2.90 (m, 3H, CH_2_ and CH oxirane), 3.85 (dd, 1H, *J* = 11.2, 6.4 Hz, OCH_2_), 4.17 (dd, 1H, *J* = 11.4, 2.6 Hz, OCH_2_). MS *m/z* (%); 266 (10.6, M^+^+1), 176 (63.2), 147 (100), 95 (48.65).

*Cyclohexyl(phenyl)methanone O-oxiran-2-yl methyl oxime* (**3b**). Yield 60%; oil; ^1^H-NMR (CDCl_3_) δ 1.25–1.70 (m, 10H, 5CH_2_ in cyclohexane), 2.15–2.41 (m, 1H, CHC=NO), 2.74–3.01 (m, 3H, CH_2_, CH oxirane), 4.11 (dd, 1H, *J* = 11.5, 6.4 Hz, OCH_2_), 4.40 (dd, 1H, *J* = 11.6, 3.0 Hz, OCH_2_), 7.55 (t, 3H, Ar-H), 7.91 (d, 2H, *J* = 7.0, 2,6 Ar-H). MS *m/z* (%); 260 (4.55, M^+^+1), 186 (100), 104 (78), 77 (61.62).

*Phenyl(Pyridin-2-yl)methanone O-oxiran-2-yl methyl oxime* (**3c**). Yield 62%; oil; ^1^H-NMR (CDCl_3_) δ 2.64–2.90 (m, 3H, CH_2_, CH oxirane), 3.91 (dd, 1H, *J* = 11.4, 6.1 Hz, OCH_2_), 4.31 (dd, 1H, *J* = 11.8, 2.9 Hz, OCH_2_), 7.44–7.68 (m, 5H, Ar-H and pyridinyl-H), 7.79 (d, 2H, *J* = 7.0, 2,6-Ar-H), 7.95 (d, 1H, *J* = 7.5, pyridinyl-H) 8.51 (d, 1H, *J* = 8.0, pyridinyl-H).

*1-(Naphthalen-2-yl)ethanone O-oxiran-2-yl methyl oxime* (**3d**). Yield 70%; M.p. 40–42 °C; ^1^H-NMR (CDCl_3_) δ 2.54–2.77 (m, 3H, CH_2_, CH oxirane), 2.95 (s, 3H, CH_3_), 3.86 (dd, 1H, *J* = 11.5, 6.5 Hz, OCH_2_), 4.17 (dd, 1H, *J* = 11.6, 2.6 Hz, OCH_2_), 7.42–7.71 (m, 7H, Ar-H). MS *m/z* (%); 242 (8.06, M^+^+1), 154 (63.2), 127 (100), 87 (48.65).

*1-(Biphenyl-4-yl)ethanone O-oxiran-2-yl methyl oxime* (**3e**). Yield 70%; M.p. 72–74 °C; ^1^H-NMR (CDCl_3_) δ 2.18 (s, 3H, CH_3_), 2.47–2.91 (m, 3H, CH_2_, CH oxirane), 3.74 (dd, 1H, *J* = 11.2, 6.6 Hz, OCH_2_), 3.96 (dd, 1H, *J* = 11.4, 2.9 Hz, OCH_2_), 7.45 (t, 3H, *J* = 8.5, 3',4',5'-Ar-H), 7.65 (d, 2H, *J* = 7.5, 2',6'-Ar-H), 7.98 (d, 2H, *J* = 8.5 Ar-H), 8.24 (d, 2H, *J* = 8.5 Ar-H).

*1-(5-Chlorothiophen-2-yl)ethanone O-oxiran-2-yl methyl oxime* (**3f**). Yield 65%; oil; ^1^H-NMR (CDCl_3_) δ 2.16 (s, 3H, CH_3_), 2.56–3.04 (m, 3H, CH_2_, CH oxirane), 4.16 (dd, 1H, *J* = 11.3, 6.5 Hz, OCH_2_), 4.30 (dd, 1H, *J* = 11.2, 2.7 Hz, OCH_2_), 7.15 (d, 1H, *J* = 8.0, 3-thiophene-H), 7.41 (d, 1H, *J* = 8.5, 4-thiophene-H).

*1,3,3-Trimethylbicyclo[2.2.1]heptan-2-one O-oxiran-2-yl methyl oxime* (**3g**). Yield 45%; oil; ^1^H-NMR (CDCl_3_) δ 1.30 (s, 6H, 2CH_3_), 1.35 (s, 3H, CH_3_), 1.52–1.70 (m, 7H, bicyclic moiety-H), 2.41–2.70 (m, 3H, CH_2_, CH oxirane), 3.75 (dd, 1H, *J* = 11.0, 6.1 Hz, OCH_2_), 3.94 (dd, 1H, *J* = 11.5, 2.4 Hz, OCH_2_). MS *m/z* (%); 224 (5.16, M^+^+1), 150 (56.62), 136 (100), 77 (35.04).

*5-Methoxy-3,4-dihydronaphthalen-1(2H)-one O-oxiran-2-yl methyl oxime* (**3h**). Yield 67%; oil; ^1^H-NMR (CDCl_3_) δ 2.17–2.91 (m, 9H, 3CH_2_ of dihydronaphthalene, CH_2_ oxirane, CH oxirane), 3.78 (s, 3H, OCH_3_), 3.85 (dd, 1H, *J* = 11.2, 6.0 Hz, OCH_2_), 4.1 (dd, 1H, *J* = 11.5, 2.7 Hz, OCH_2_), 7.14 (d, 1H, *J* = 8.0, 6-Ar-H), 7.35 (t, 1H, *J* = 8.5, 7-Ar-H), 7.56 (d, 1H, *J* = 8.5, 8-Ar-H).

#### 3.1.2. General Procedure for Synthesis of *O*-(3-Alkylamino-2-hydroxypropyl)oxime Derivatives **4a**–**z**

A mixture of the appropriate oxirane **3a–j** (0.01 mol) and the appropriate amine (isopropylamine, *tert*-butylamine or homoveratrylamine, 0.04 mol) in anhydrous toluene (10 mL) was heated in sealed tube at 120 °C for 24 h (TLC). After cooling, the solution was evaporated under reduced pressure to give an oily residue. The obtained product was dissolved in diethylether, and treated with oxalic acid (0.45 g, 0.005 mol) in acetone or ethereal HCl to give either the oxalate or hydrochloride salt. The separated solid was collected by filtration, washed with diethyl ether, dried and crystallized from ethanol.

*Dicyclohexylmethanone O-2-hydroxy-3-(isopropylamino)propyl oxime*, *hydrogen oxalate* (**4a**). Yield 63%; M.p. 123–125 °C; ^1^H-NMR (free base in CDCl_3_) δ 1.06 (d, 6H, *J* = 6.5, 2CH_3_), 1.52–1.77 (m, 20H, 10CH_2_ cyclohexane), 2.42–2.65 (m, 2H, 2CHC=NO), 2.95–3.12 (m, 3H, CH_2_N, CH(CH_3_)_2_), 3.4–3.7 (m, 3H, CHOH, OH and NH exchangeable with D_2_O), 3.95 (dd, 1H, *J* = 10.8, 6.4 Hz, OCH_2_), 4.15 (dd, 1H, *J* = 11.2, 2.8 Hz, OCH_2_). MS *m/z* (%); 326 (6.19, M^+^+2), 325 (6.82, M^+^+1), 210 (41.72), 98 (100), 83 (34.55), 72 (81.95).

*Dicyclohexylmethanone O-3-(tert-butylamino)-2-hydroxypropyl oxime*, *hydrogen oxalate* (**4b**). Yield 55%; M.p. 117–119 °C; ^1^H-NMR (free base in CDCl_3_) δ 1.46 (s, 9H, 3CH_3_), 1.59–1.72 (m, 20H, 10CH_2_ cyclohexane), 2.41–2.55 (m, 2H, CHC=NO), 2.81–2.98 (m, 2H, CH_2_NH), 3.35–3.54 (m, 3H, CHOH, OH and NH exchangeable with D_2_O), 4.12 (dd, 1H, *J* = 11.2, 6.2 Hz, OCH_2_), 4.26 (dd, 1H, *J* = 11.8, 2.1 Hz, OCH_2_). MS *m/z* (%); 340 (9.9, M^+^+2), 339 (5.04, M^+^+1), 225 (38.09), 113 (100), 86 (10.24), 72 (76.36).

*Dicyclohexylmethanone O-3-(3,4-dimethoxyphenethylamino)-2-hydroxypropyl* *oxime, hydrogen oxalate* (**4c**). Yield 50%; M.p. 174–176 °C; 1H-NMR (free base in CDCl_3_) δ 1.5–1.8 (m, 20H, 10CH_2_ cyclohexane), 2.4–2.9 (m, 8H, 2CHC=NO, CH_2_NHCH_2_CH_2_), 3.50–3.71 (m, 3H, CHOH, OH and NH exchangeable with D_2_O), 3.97 (s, 6H, 2OCH_3_), 4.08 (dd, 1H, *J* = 10.8, 6.0 Hz, OCH_2_), 4.24 (dd, 1H, *J* = 11.5, 2.5 Hz, OCH_2_), 6.82 (d, 1H, *J* = 9.0, Ar-H), 7.01 (s, 1H, Ar-H), 7.21 (d, 1H, *J* = 8.5, Ar-H).

*Cyclohexyl(phenyl)methanone O-2-hydroxy-3-(isopropylamino)propyl oxime, hydrochloride* (**4d**). Yield 70%; M.p. 157–159 °C; ^1^H-NMR (free base in CDCl_3_) δ 1.15 (d, 6H, *J* = 6.5, 2CH_3_), 1.52–1.77 (m, 10H, 5CH_2_ cyclohexane), 2.32–2.41 (m, 1H, CHC=NO), 2.77–2.89 (m, 3H, CH_2_N, CH(CH_3_)_2_), 3.45–3.62 (m, 3H, CHOH, OH and NH exchangeable with D_2_O), 3.98 (dd, 1H, *J* = 11.2, 6.1 Hz, OCH_2_), 4.12 (dd, 1H, *J* = 11.6, 2.7 Hz, OCH_2_), 7.45 (m, 5H, Ar-H). MS *m/z* (%); 320 (6.94, M^+^+2), 319 (3.75, M^+^+1), 188 (68.77), 104 (70.80), 72 (100).

*Cyclohexyl(phenyl)methanone O-3-(tert-butylamino)-2-hydroxypropyl oxime, hydrochloride* (**4e**). Yield 55%; M.p. 107–109 °C; ^1^H-NMR (free base in DMSO-*d*_6_) δ 1.21 (s, 9H, 3CH_3_), 1.47–1.68 (m, 10H, 5CH_2_ cyclohaxane), 2.45–2.62 (m, 1H, CHC=NO), 3.1–3.4 (m, 2H, CH_2_N), 3.44–3.68 (m, 3H, CHOH, OH and NH exchangeable with D_2_O), 3.88 (dd, 1H, *J* = 11.0, 6.5 Hz, OCH_2_), 4.19 (dd, 1H, *J* = 11.3, 2.5 Hz, OCH_2_), 7.55 (m, 5H, Ar-H). MS *m/z* (%); 334 (7.80, M^+^+2), 333 (5.10, M^+^+1), 146 (50.15), 172 (73.08), 72 (100).

*Cyclohexyl(phenyl)methanone O-3-(3,4-dimethoxyphenethyl amino)-2-hydroxypropyl oxime*, *hydrogen oxalate* (**4f**). Yield 48%; M.p. 140–142 °C; ^1^H-NMR (free base in CDCl_3_) δ 1.40–1.70 (m, 10H, 5CH_2_ cyclohaxane), 2.38–2.65 (m, 7H, CHC=NO, CH_2_NHCH_2_CH_2_), 3.40–3.65 (m, 3H, CHOH, OH and NH exchangeable with D_2_O), 3.8 (s, 6H, 2OCH_3_), 4.00 (dd, 1H, *J* = 11.5, 6.2 Hz, OCH_2_), 4.18 (dd, 1H, *J* = 11.6, 2.6 Hz, OCH_2_), 6.9 (d, 1H, *J* = 8.5, Ar-H homoveratryl), 7.2 (d, 1H, *J* = 8.5, Ar-H homoveratryl), 7.2 (s, 1H, Ar-H homoveratryl), 7.6 (m, 5H, Ar-H). MS *m/z* (%); 442 (10.12, M^+^+2), 441 (9.33, M^+^+1), 289 (27.80) 186 (41.90), 104 (100).

*Phenyl(pyridin-2-yl)methanone O-2-hydroxy-3-(isopropylamino)propyl oxime, hydrochloride* (**4g**). Yield 69%; M.p. 191–193 °C; ^1^H-NMR (free base in DMSO-*d*_6_) δ 1.44 (d, 6H, *J* = 6.5, 2CH_3_), 2.95–3.12 (m, 3H, CH_2_N, CH(CH_3_)_2_), 3.6–3.8 (m, 3H, CHOH, OH and NH exchangeable with D_2_O), 4.05 (dd, 1H, *J* = 11.5, 6.0 Hz, OCH_2_), 4.24 (dd, 1H, *J* = 11.0, 2.5 Hz, OCH_2_), 7.4–7.7 (m, 5H, 3,4,5-Ar-H and 3,5- pyridinyl-H), 7.9–8.1 (m, 3H, 2,6-Ar-H, 4-pyridinyl-H), 8.5 (d, 1H, *J* = 8.0, 6-pyridinyl-H). MS *m/z* (%); 315 (0.13, M^+^+2), 314 (0.55, M^+^+1), 313 (0.20, M^+^), 198 (100), 181 (40.71), 72 (44.96). 

*Phenyl(pyridin-2-yl)methanone O-3-(tert-butylamino)-2-hydroxypropyl oxime, hydrochloride* (**4h**). Yield 55%; M.p. 180–182 °C; ^1^H-NMR (free base in DMSO-*d*_6_) δ 1.27 (s, 9H, 3CH_3_), 2.94–3.08 (m, 3H, CH_2_N), 3.5–3.8 (m, 3H, CHOH, OH and NH exchangeable with D_2_O), 4.14 (dd, 1H, *J* = 11.5, 6.2 Hz, OCH_2_), 4.33 (dd, 1H, *J* = 11.6, 2.8 Hz, OCH_2_), 7.6–7.8 (m, 5H, 3,4,5-Ar-H and 3,5- pyridinyl-H), 8.0–8.2 (m, 3H, 2,6-Ar-H, 4-pyridinyl-H), 8.5 (d, 1H, *J* = 8, 6-pyridinyl-H). MS *m/z* (%); 329 (5.38, M^+^+2), 328 (1.35, M^+^+1), 198 (100), 183 (70.91), 86 (46.49), 78 (42.53).

*Phenyl (pyridin-2-yl)methanone O-3-(3,4-dimethoxyphenethyl amino)-2-hydroxypropyl oxime*, *hydrogen oxalate* (**4i**). Yield 50%; M.p. 177–179 °C; ^1^H-NMR (free base in CDCl_3_) δ 2.4–2.9 (m, 6H, CH_2_NHCH_2_CH_2_), 3.50–3.85 (m, 3H, CHOH, OH and NH exchangeable with D_2_O), 3.97 (s, 6H, 2OCH_3_), 4.10 (dd, 1H, *J* = 11.5, 6.0 Hz, OCH_2_), 4.22 (dd, 1H, *J* = 11.0, 2.8 Hz, OCH_2_), 6.8 (d, 2H, 5,6-Ar-H homoveratryl), 7.1 (s, 1H, 2-Ar-H homoveratryl), 7.4–7.7 (m, 5H, 3,4,5-Ar-H and 3,5- pyridinyl-H), 7.9 (m, 3H, 2,6-Ar-H, 4-pyridinyl-H), 8.5 (d, 1H, *J* = 8.5, 6-pyridinyl-H).

*1-(Naphthalen-2-yl)ethanone O-2-hydroxy-3-(isopropylamino)propyl oxime, hydrochloride* (**4j**). Yield 67%; M.p. 117–119 °C; ^1^H-NMR (free base in CDCl_3_) δ 1.61 (d, 6H, *J* = 6.5, 2CH_3_), 2.20 (s, 3H, CH_3_), 2.7–2.9 (m, 2H, CH_2_NH), 3.44–3.65 (m, 3H, CHOH, OH and NH exchangeable with D_2_O), 4.42 (dd, 1H, *J* = 11.6, 6.4 Hz, OCH_2_), 4.57 (dd, 1H, *J* = 11.2, 2.4 Hz, OCH_2_), 7.65 (t, 2H, *J* = 8.0, 6,7-Ar-H), 8.06 (d, 4H, *J* = 7.5, 3,4,5,8- Ar-H), 8.45 (s, 1H, 1-Ar-H). MS *m/z* (%); 300 (1.60, M^+^), 168 (6.54), 127 (21.09), 73 (91.92), (100), 60 (55.21).

*1-(Naphthalen-2-yl)ethanone O-3-(tert-butylamino)-2-hydroxypropyl oxime, hydrochloride* (**4k**). Yield 59%; M.p. 120–122 °C; ^1^H-NMR (free base in CDCl_3_) δ 1.18 (s, 9H, 3CH_3_), 2.38 (s, 3H, 3CH_3_), 3.0–3.4 (m, 2H, CH_2_NH), 3.4–3.75 (m, 3H, CHOH, OH and NH exchangeable with D_2_O), 4.24 (dd, 1H, *J* = 11.3, 6.2 Hz, OCH_2_), 4.38 (dd, 1H, *J* = 11.8, 2.2 Hz, OCH_2_), 7.56 (t, 2H, *J* = 8.5 6,7-Ar-H), 8 (d, 4H, *J* = 7.5, 3,4,5,8- Ar-H), 8.4 (s, 1H, 1-Ar-H). MS *m/z* (%); 316 (9.25, M^+^+2), 315 (7.18, M^+^+1), 167 (84.11), 127 (60.13), 86 (100), 58 (43.86).

*1-(Naphthalen-2-yl)ethanone O-3-(3,4-dimethoxyphenethyl amino)-2-hydroxypropyl oxime, hydrogen oxalate* (**4l**). Yield 50%; M.p. 181–183 °C; ^1^H-NMR (free base in CDCl_3_) δ 2.10 (s, 3, CH_3_), 3.1–3.4 (m, 6H, CH_2_NHCH_2_CH_2_), 3.3.75 (m, 3H, CHOH, OH and NH exchangeable with D_2_O), 3.8 (s, 6H, 2OCH_3_), 4.15 (dd, 1H, *J* = 11.5, 6.2 Hz, OCH_2_), 4.30 (dd, 1H, *J* = 11.6, 2.6 Hz, OCH_2_), 6.8 (d, 2H, *J* = 8.0, 5,6-Ar-H homoveratryl), 7.17 (s, 1H, 2-Ar-H homoveratryl), 7.65 (t, 2H, *J* = 8.5, 6,7-Ar-H), 8.14 (d, 4H, *J* = 7.5, 3,4,5,8-Ar-H), 8.62 (s, 1H, 1-Ar-H). MS *m/z* (%); 424 (12.07, M^+^+2), 423 (9.11, M^+^+1), 181 (11.91), 152 (100), 137 (28.19).

*1-(Biphenyl-4-yl)ethanone O-2-hydroxy-3-(isopropylamino)propyl oxime*, *hydrogen oxalate* (**4m**). Yield 60%; M.p. 169–171 °C; ^1^H-NMR (free base in DMSO-*d*_6_) δ 1.21 (d, 6H, *J* = 6.5, 2CH_3_), 2.16 (s, 3H, CH_3_C), 2.95–3.12 (m, 3H, CH_2_N, CH(CH_3_)), 3.45–3.72 (m, 3H, CHOH, OH and NH exchangeable with D_2_O), 3.89 (dd, 1H, *J* = 10.9, 6.1 Hz, OCH_2_), 4.29 (dd, 1H, *J* = 11.0, 2.5 Hz, OCH_2_), 7.2–7.4 (m, 3H, 3',4',5'-Ar-H), 7.56 (d, 2H, *J* = 8.0, 2',6'-Ar-H), 7.84 (d, 4H, *J* = 8.5, 2,3,5,6-Ar-H).

*1-(Biphenyl-4-yl)ethanone O-3-(tert-butylamino)-2-hydroxypropyl oxime, hydrochloride* (**4n**). Yield 52%; M.p. 165–167 °C;^ 1^H NMR (free base in DMSO-*d*_6_) δ 1.21 (s, 9H, 3CH_3_), 2.16 (s, 3H, CH_3_C), 2.95–3.12 (m, 3H, CH_2_N, CH(CH_3_)), 3.45–3.72 (m, 3H, CHOH, OH and NH exchangeable with D_2_O), 3.89 (dd, 1H, *J* = 10.9, 6.1 Hz, OCH_2_), 4.29 (dd, 1H, *J* = 11.0, 2.5 Hz, OCH_2_), 7.2–7.4 (m, 3H, 3',4',5'-Ar-H), 7.56 (d, 2H, *J* = 8.0, 2',6'-Ar-H), 7.84 (d, 4H, *J* = 8.5, 2,3,5,6-Ar-H). MS *m/z* (%); 342 (8.39, M^+^+2), 341 (7.71, M^+^+1), 193 (69), 153 (39.43), 86 (100). 

*1-(Biphenyl-4-yl)ethanone O-3-(3,4-dimethoxyphenethylamino)-2-hydroxypropyl oxime, hydrochloride* (**4o**). Yield 48%; M.p. 157–159 °C; ^1^H-NMR (free base in CDCl_3_) δ 2.2 (s, 3H, CH_3_), 2.85–3.1 (m, 6H, CH_2_NHCH_2_CH_2_), 3.45–3.72 (m, 3H, CHOH, OH and NH exchangeable with D_2_O), 3.85 (s, 6H, 2OCH_3_) 4.02 (dd, 1H, *J* = 11.2, 6.2 Hz, OCH_2_), 4.22 (dd, 1H, *J* = 11.5, 2.2 Hz, OCH_2_), 6.85 (d, 2H, *J* = 8.0, 5,6-Ar-H homoveratryl), 7.25 (s, 1H, 2-Ar-H homoveratryl), 7.3–7.5 (m, 3H, 3',4',5'-Ar-H), 7.59 (d, 2H, *J* = 8.0, 2',6'-Ar-H), 7.88 (d, 4H, *J* = 8.5, 2,3,5,6-Ar-H). MS *m/z* (%); 450 (11.9, M^+^+2), 449 (6.12, M^+^+1), 297 (34.08), 194 (100).

*1-(5-Chlorothiophen-2-yl)ethanone O-2-hydroxy-3-(isopropyl amino)propyl oxime, hydrochloride* (**4p**). Yield 70%; M.p. 145–147 °C; ^1^H-NMR (free base in DMSO-*d*_6_) δ 1.03 (d, 6H, *J* = 7.0, 2CH_3_), 2.10 (s, 3H, CH_3_C=NO), 2.85–3.06 (m, 3H, CH_2_N, CH(CH_3_)_2_)), 3.5–3.7 (m, 3H, CHOH, OH and NH exchangeable with D_2_O), 4.11 (dd, 1H, *J* = 11.5, 6.2 Hz, OCH_2_), 4.39 (dd, 1H, *J* = 11.5, 2.2 Hz, OCH_2_), 7.17 (d, 1H, *J* = 4.0, 3-thiophene-H), 7.35 (d, 1H, *J* = 4.1, 4-thiophene -H).

*1-(5-Chlorothiophen-2-yl)ethanone O-3-(tert-butylamino)-2-hydroxypropyl oxime, hydrochloride* (**4q**). Yield 62%; M.p. 152–154 °C;^ 1^H-NMR (free base in DMSO-*d*_6_) δ 1.33 (s, 9H, 3CH_3_), 2.21 (s, 3H, CH_3_C=NO), 2.77–2.96 (m, 2H, CH_2_N) 3.47–3.66 (m, 3H, CHOH, OH and NH exchangeable with D_2_O), 4.08 (dd, 1H, *J* = 11.4, 6.5 Hz, OCH_2_), 4.38 (dd, 1H, *J* = 11.3, 2.8 Hz, OCH_2_), 6.98 (d, 1H, *J* = 4.2, 3-thiophene-H), 7.37 (d, 1H, *J* = 4.0, 4-thiophene-H). MS *m/z* (%); 306 (2.06, M^+^+2), 305 (1.45, M^+^+1), 160 (100), 145 (82.20), 72 (84.98).

*1-(5-Chlorothiophen-2-yl)ethanone O-3-(3,4-dimethoxyphenethylamino)-2-hydroxypropyl oxime, hydrochloride* (**4r**). Yield 52%; M.p. 148–150 °C; ^1^H-NMR (free base in DMSO-*d*_6_) δ 2.19 (s, 3H, CH_3_C=NO), 2.79–3.05 (m, 6H, CH_2_NHCH_2_CH_2_), 3.4–3.6 (m, 3H, CHOH, OH and NH exchangeable with D_2_O), 4.0 (dd, 1H, *J* = 11.0, 6.0 Hz, OCH_2_), 4.32 (dd, 1H, *J* = 11.5, 2.6 Hz, OCH_2_), 6.88 (d, 2H, *J* = 8.0, 5,6-Ar-H homoveratryl), 7.17 (d, 1H, *J* = 4.0, 3-thiophene-H), 7.29 (s, 1H, 2-Ar-H homoveratryl), 7.39 (d, 1H, *J* = 4.01, 4-thiophene -H). MS *m/z* (%); 413 (3.22, M^+^), 261 (77.44), 158 (100), 73 (13.79).

*1,3,3-Trimethylbicyclo[2.2.1]heptan-2-one O-2-hydroxy-3-(isopropylamino)propyl oxime, hydrochloride* (**4s**). Yield 55%; M.p. 127–129 °C; ^1^H-NMR (free base in CDCl_3_) δ 1.14 (d, 6H, *J* = 6.5, 2CH_3_), 1.25 (s, 6H, 2CH_3_), 1.32 (s, 3H, CH_3_), 1.44–1.81 (m, 7H, CH, 3CH_2_ cyclic moiety), 2.7–3.0 (m, 2H, CH_2_N), 3.33–3.71 (m, 3H, CHOH, OH and NH exchangeable with D_2_O), 4.11 (dd, 1H, *J* = 11.2, 6.5 Hz, OCH_2_), 4.31 (dd, 1H, *J* = 11.7, 2.5 Hz, OCH_2_). MS *m/z* (%); 284 (11.25, M^+^+2), 167 (12.71), 81 (100), 69 (36.25).

*1,3,3-Trimethylbicyclo[2.2.1]heptan-2-one O-3-(tert-butylamino)-2-hydroxypropyl oxime*, *hydrogen oxalate* (**4t**). Yield 52%; M.p. 115–117 °C; ^1^H-NMR (free base in CDCl_3_) δ 1.12 (s, 9H, 3CH_3_), 1.25 (s, 6H, 2CH_3_), 1.34 (s, 3H, CH_3_), 1.4–1.8 (m, 7H, CH, 3CH_2_ cyclic moiety), 2.9–3.4 (m, 2H, CH_2_N), 3.65–3.9 (m, 3H, CHOH, OH and NH exchangeable with D_2_O), 3.88 (dd, 1H, *J* = 11.5, 6.5 Hz, OCH_2_), 4.21 (dd, 1H, *J* = 11.0, 2.8 Hz, OCH_2_).

*1,3,3-Trimethylbicyclo[2.2.1]heptan-2-one O-3-(3,4-dimethoxyphenethylamino)-2-hydroxypropyl oxime*, *hydrogen oxalate* (**4u**). Yield 39%; M.p. 140–142 °C; ^1^H-NMR (free base in DMSO-*d*_6_) δ 1.22 (s, 6H, 2CH_3_), 1.31 (s, 3H, CH_3_), 1.5–1.78 (m, 7H, CH, 3CH_2_ in cyclic moiety), 2.4–2.9 (m, 6H, CH_2_NHCH_2_CH_2_), 3.61–3.81 (m, 3H, CHOH, OH and NH exchangeable with D_2_O), 3.97 (s, 6H, OCH_3_), 4.10 (dd, 1H, *J* = 11.6, 6.4 Hz, OCH_2_), 4.22 (dd, 1H, *J* = 11.4, 2.5 Hz, OCH_2_), 6.9 (d, 2H, *J* = 8.5, 5,6-Ar-H), 7.15 (s, 1H, 2-Ar-H). MS *m/z* (%); 406 (9.17, M^+^+2), 405 (7.45, M^+^+1), 253 (26.99), 151 (50.87), 104 (88.50), 81 (100). 

*5-Methoxy-3,4-dihydronaphthalen-1(2H)-one O-2-hydroxy-3-(isopropylamino)propyl oxime*, *hydrogen oxalate* (**4v**). Yield 76%; M.p. 180–182 °C; ^1^H-NMR (free base in CDCl_3_) δ 1.21 (d, 6H, *J* = 6.5, 2CH_3_), 2.22–2.61 (m, 6H, 3CH2 cyclic structure), 2.8–3.1 (m, 3H, CH_2_NCH), 3.5–3.7 (m, 3H, CHOH, OH and NH exchangeable with D_2_O), 3.95 (dd, 1H, *J* = 11.5, 6.4 Hz, OCH_2_), 4.14 (dd, 1H, *J* = 11.6, 2.6 Hz, OCH_2_), 4.19 (s, 3H, OCH_3_), 7.11 (d, 1H, *J* = 9.0, 6-Ar- H), 7.36 (t, 1H, *J* = 8.5, 7-Ar-H), 7.55 (d, 1H, *J* = 8.5, 8-Ar-H). MS *m/z* (%); 308 (6.55, M^+^+2), 307 (3.45, M^+^+2), 248 (35), 174 (100).

*5-Methoxy-3,4-dihydronaphthalen-1(2H)-one O-3-(tert-butylamino)-2-hydroxypropyl oxime*, *hydrogen oxalate* (**4w**). Yield 69%; M.p. 177–179 °C; ^1^H-NMR (free base in CDCl_3_) δ 1.21 (s, 9H, 3CH_3_), 2.03–2.81 (m, 6H, 3CH_2_ cyclic structure), 3.1–3.4 (m, 2H, CH_2_N), 3.5–3.7 (m, 3H, CHOH, OH and NH exchangeable with D_2_O), 3.89 (dd, 1H, *J* = 11.5, 6.2 Hz, OCH_2_), 4.08 (dd, 1H, *J* = 11.6, 2.4 Hz, OCH_2_), 4.15 (s, 3H, OCH_3_), 7.07 (d, 1H, *J* = 9.0, 6-Ar-H), 7.31 (t, 1H, *J* = 8.5, 7-Ar-H), 7.52 (d, 1H, *J* = 8.5, 8-Ar-H).

*5-Methoxy-3,4-dihydronaphthalen-1(2H)-one O-3-(3,4-dimethoxyphenethylamino)-2-hydroxypropyl oxime*, *hydrogen oxalate* (**4x**). Yield 58%; M.p. 160–162 °C; ^1^H-NMR (free base in DMSO-*d*_6_) δ 2.1–2.9 (m, 12H, CH_2_NHCH_2_CH_2_, 3CH_2_ cyclic structure), 3.51–3.70 (m, 3H, CHOH, OH and NH exchangeable with D_2_O), 3.82 (s, 6H, 2OCH_3_), 3.88 (s, 3H, OCH_3_), 4.11 (dd, 1H, *J* = 11.1, 6.4 Hz, OCH_2_), 4.27 (dd, 1H, *J* = 11.3, 3.0 Hz, OCH_2_), 6.79 (d, 2H, *J* = 8.5, 5,6-Ar-H homoveratryl), 7.0 (s, 1H, 2-Ar-H homoveratryl), 7.25 (d, 1H, *J* = 8.0, 6-Ar-H), 7.47 (t, 1H, *J* = 9.0, 7-Ar-H), 7.62 (d, 1H, *J* = 8.0, 8-Ar-H). 

*Diphenylmethanone O-3-(3,4-dimethoxyphenethylamino)-2-hydroxypropyl oxime, hydrochloride* (**4y**). Yield 52%; M.p. 166–168 °C; ^1^H-NMR (free base in DMSO-*d*_6_) δ 2.58–2.96 (m, 6H, CH_2_NHCH_2_CH_2_), 3.6–3.9 (m, 3H, CHOH, OH and NH exchangeable with D_2_O), 3.95 (s, 6H, 2OCH_3_), 4.16 (dd, 1H, *J* = 10.8, 5.9 Hz, OCH_2_), 4.28 (dd, 1H, *J* = 11.4, 3.4 Hz, OCH_2_), 6.67 (d, 2H, *J* = 8.5, 5,6-Ar-H homoveratryl), 7.18 (s, 1H, 2-Ar-H homoveratryl), 7.60–8.02 (m, 10H, Ar-H). MS *m/z* (%); 436 (5.85, M^+^+2), 435 (6.79, M^+^+1), 152 (100), 137 (26.50). 

*1-Phenylethan-1-one O-3-(3,4-dimethoxyphenethylamino)-2-hydroxypropyl oxime, hydrochloride* (**4z**). Yield 56%; M.p. 138–141 °C; ^1^H-NMR (free base in DMSO-*d*_6_) δ 1.91 (s, 3H, CH_3_), 2.4–2.9 (m, 6H, CH_2_NHCH_2_CH_2_), 3.6–3.9 (m, 3H, CHOH, OH and NH exchangeable with D_2_O), 3.90 (s, 6H, 2OCH_3_), 4.05 (dd, 1H, *J* = 11.2, 6.4 Hz, OCH_2_), 4.25 (dd, 1H, *J* = 11.9, 2.7 Hz, OCH_2_), 6.87 (d, 2H, 5,6-Ar-H homoveratryl), 7.15 (s, 1H, 2-Ar-H homoveratryl), 7.53 (t, 3H, *J* = 8, Ar-H), 7.9 (d, 2H, *J* = 8.5, Ar-H). MS *m/z* (%); 374 (5.97, M^+^+2), 375 (7.45, M^+^+1), 165 (19.97), 118 (100), 77 (29.65).

### 3.2. Molecular Docking Methodology

Automated docking simulations were conducted with the Molegro Virtual Docker 2007 (MVD 2007.2.2.5–Aug 27, 2007 [win32]) fully functional free trial version with time limiting license [20] 3D Molecular structures and energy minimization were carried out by free version of Marvinsketch 4.1.13 from Chemaxon Ltd. (Budapest, Hungary) [28]. 3D crystal structures of β_1_ and β_2_-adrenergic receptors were obtained from Protein Data Bank (PDB); entries 2VT4 and 2R4R, respectively [18,19,29]. All the molecular modeling studies were carried out on a PC equipped with an Intel Celeron 1.2 GHz processor, and 320 MB memory running the Windows XP operating system.

The docking study was performed, in the present investigation, following a general procedure for docking [30,31]. The coordinates of the crystal structures adrenergic receptors were imported into Molegro Virtual Docker. Receptor structures were checked for missing atoms, bonds and contacts. The ligand molecules were constructed in 2D form then converted to 3D form and energy minimized by Marvinsketch. Energy minimized conformers were imported into Molegro Virtual Docker. The dimensions of the docking constraint were manipulated so as to accommodate all the amino acid residues present in the active site (radius 12 Ǻ). The ligands were manually docked into the binding cavity of β_2_ adrenergic receptor, guided by all the amino acids present in the active site, and snapshots were recorded. Figure 2a,b represent the docking snapshots of compounds **4o** and **4q** with the β_1_ and β_2_-adrenergic receptors, respectively, showing the forces of interaction. In this study the MolDock score function and hydrogen bond interactions between tested compounds and the target receptors were used to compare between the tested compounds and reference drugs. The MolDock score is a new technique for high-accuracy molecular docking. It is an extension of the piecewise linear potential (PLP) [32,33] including new hydrogen bonding and electrostatic terms. To further improve docking accuracy, a re-ranking scoring function is introduced, which identifies the most promising docking solution from the solutions obtained by the docking algorithm [34]. The lower MolDock score means better ligand-receptor interactions. The MolDock scores and hydrogen bond were recorded (Table 3).

### 3.3. Biological Evaluation

Epinephrine hydrochloride injection (0.25 mg/1 mL) was obtained from Misr Co. Cairo, Egypt. Propranolol hydrochloride (Inderal injection) B.P. 0.1%, was obtained from AstraZeneca Limited (Cairo, Egypt). Acetylcholine chloride and salbutamol sulfate were purchased from Sigma-Aldrich chemical Co. All other chemicals used in this study were of finest analytical grade, and obtained from El-Nasr Pharmaceutical Chemicals Company. Tested compounds were dissolved in distilled water or 20% ethanol, just before use, in 10^−5^ mole concentration. Adult male Hartley guinea pigs weighing 350–500 g were used. Animals were maintained under standard conditions of temperature with regular 12 h light/dark cycles and allowed free access to standard laboratory food and water. Biological evaluation using animals was performed in accordance with the Ethics Committee of faculty of Pharmacy Mansoura University.

#### 3.3.1. *In Vitro* Screening for β_1_-Adrenoceptor Activity in Isolated Guinea Pig Atria

Guinea pigs were anaesthetized by diethyl ether. The chest was opened and the heart was quickly excised and placed in oxygenated Ringer Locke solution of the following composition (g/L): NaCl (9), KCl (0.42), CaCl_2_ (0.24), NaHCO_3_ (0.15) and glucose (1). Atria were dissected away from the rest of the heart, freed from connective tissue and suspended in 50 mL organ bath containing Ringer Locke solution. This solution is thermoregulated at 37 °C and continuously bubbled with pure oxygen. The extremities of the strip, consisting of both atria, were tied with a thread. The first thread was used to tie the organ to a muscle holder fixed in place in a muscle chamber of an isolate organ bath; the second thread was used to connect atria to an isomeric force-displacement transducer (model 50–7905, Harvard Apparatus Inc, South Natick, MA, USA) situated above the muscle chamber and connected to a two-channel oscillograph (model 50–8622, Harvard Apparatus). Atria spontaneously beating were loaded with 0.5 g and were allowed to adjust to the bath conditions for at least 30 min prior to the experiment, and the Ringer Locke solution was changed at 10 min intervals. Epinephrine (43 µL of epinephrine vial; 10^−6^ mol) was applied and allowed to act till the response was fully developed. The atria were washed with Ringer Locke solution. Test compound (10^−5^mol, 2.5–4.5 mg/L) was applied and incubated for 5 min in the organ bath to test and evaluate any possible agonistic activity on the β_1_-adrenergic receptor. Then, epinephrine (43 µL of epinephrine vial; 10^−6^ mol) was added in the organ bath in order to evaluate any competitive antagonistic activity of the test compound to epinephrine on the β_1_-adrenergic receptor. Propranolol (10^−5^ mol) was used as reference drug.

#### 3.3.2. *In Vitro* Screening for β_2_-Adrenoceptor Activity in Isolated Guinea Pig Trachea

Guinea pig was anaesthetized by diethylether; the trachea was carefully isolated and immersed in oxygenated Krebs-Hanseleit solution of the following composition (g/L): NaCl (6.9), KCl (0.35), KH_2_PO_4_ (0.16), NaHCO_3_ (2.1), MgSO_4_ (0.29), anhydrous CaCl_2_ (0.28) and glucose (2). The tracheal preparation was cautiously cleaned of unnecessary adipose and connective tissues. Subsequently, the tracheal cartilage containing smooth muscles was cut into zig-zag strips according to Emmerson and Mackay method [35]. Zig-zag tracheal strips were tied at each end with a thread. The first thread was used to tie the organ to a muscle holder fixed in place in a muscle chamber of an isolate organ bath; the second thread was used to connect trachea to an isomeric force-displacement transducer (model 50–7905, Harvard Apparatus) situated above the muscle chamber and connected to a two-channel oscillograph (model 50–8622, Harvard Apparatus). The organ bath contains 50 mL of Krebs-Hanseleit solution at 32 °C and gassed with pure oxygen. The zig-zag tracheal strips were loaded with 1 g and were left to stabilize for 1 h, and the Krebs-Hanseleit solution was changed at 15 min intervals. The zig-zag tracheal strips were precontracted with acetylcholine chloride (0.9 mg/50 mL; 10^−4^ mol) and allowed to act till the response was fully developed. The test compound (10^−5^ mol) was then added and incubated for 20 min in the organ bath to evaluate its agonistic activity on β_2_-adrenergic receptor. Salbutamol sulfate (10^−5^ mol) was then added in organ bath in the presence of the test compound in order to evaluate any blocking activity of the test compound to β_2_- adrenergic receptor. Propranolol (10^−5^ mol) was used as reference drug.

## 4. Conclusions

In this study, a series of oxime ether derivatives **4a**–**z** were designed and successfully synthesized in convenient steps. Selected compounds were tested for their biological activities against *in vitro* β_1_- and β_2_-adrenergic receptors and also examine their binding energies with 3D crystal structures of β_1_ and β_2_ receptors by doing molecular docking analysis. These findings confirm the significance of a homoveratrylamine portion for β_1_ receptors and also indicate the importance of the presence of a chlorothiophene moiety in the hydrophobic region for best complementarity with β_2_ receptors.

## Supplementary Materials

Supplementary materials can be accessed at: http://www.mdpi.com/1420-3049/19/3/3417/s1.
