# Supplementary material for: Synthesis, Docking Study and β-Adrenoceptor Activity of Some New Oxime Ether Derivatives
Source: Molecules. 2014 Mar 20;19(3):3417–35. doi: 10.3390/molecules19033417 (PMC6271474; doi:10.3390/molecules19033417)
Supplement: Supplementary file 1 [file molecules-19-03417-s001.pdf]

Table S1. The different forces of interaction between the compounds and the active site of the receptor.

<table>
<tr><th>Compound name/ structure/
docking snapshot</th><th>Forces involved in the ligand-receptor
interaction</th></tr>
<tr><td>

Epinephrine

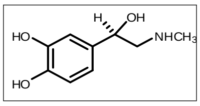

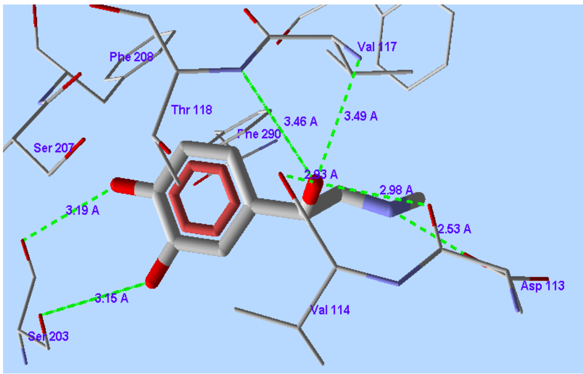

</td><td>

Hydrogen bonding interactions:
-*p*-Hydroxyl group of epinephrine with carbonyl group of Ser 203 (3.19 Å)
-*m*-Hydroxyl group with hydroxyl group of Ser 203 (3.15 Å)
-β-Hydroxyl group with peptide carbonyl group of Asp 113 (2.98 Å), carbonyl group of Val 114 (2.93 Å), amino group of Thr 118 (3.46 Å) and with amino group of Val 117 (3.49 Å).
-Terminal amino group with carbonyl of free carboxylic group of Asp 113 (2.53 Å)
Ionic interaction:
Terminal amino group with carboxylate ion of Asp 113.
Hydrophobic interactions:
Phenyl ring and Phe 290.

</td></tr>
<tr><td>

Salbutamol

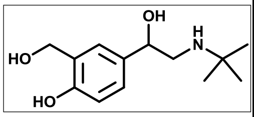

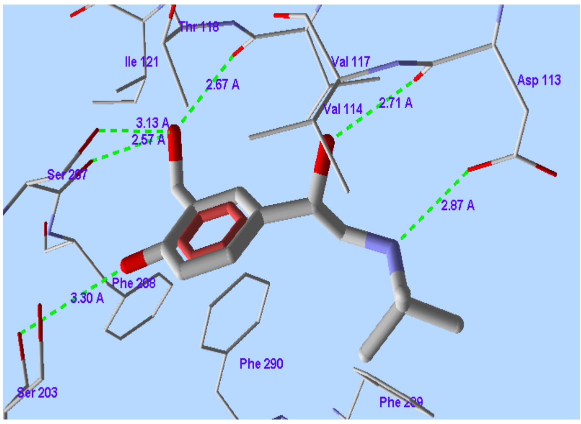

</td><td>

Hydrogen bonding interactions:
-*p*-Hydroxyl group of salbutamol with carbonyl group of Ser 203 (3.30 Å)
-*m*-Hydroxymethyl group with hydroxyl group of Ser 207 (3.13 Å), carbonyl group of Val 117 (2.67 Å) and with carbonyl group of Ser 207 (2.57 Å).
-β-Hydroxyl group with peptide carbonyl group of Asp 113 (2.71 Å)
-Terminal amino group with carbonyl of free carboxylic group of Asp 113 (2.87 Å)
Ionic interaction:
Terminal amino group with carboxylate ion of Asp 113.
Hydrophobic interactions:
Phenyl ring of salbutamol and Phe 290.

</td></tr>
</table>

Table S1. *Cont.*

Compound name/ structure/ docking snapshot	Forces involved in the ligand-receptor interaction
**Propranolol** 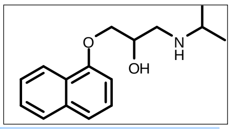 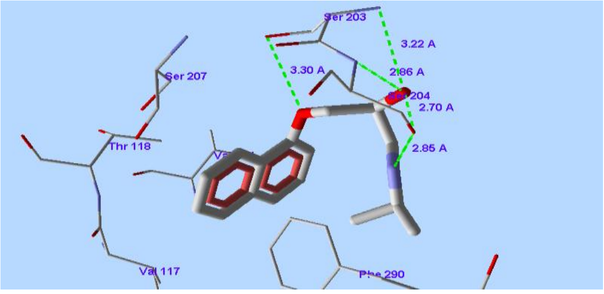	**Hydrogen bonding interactions:**<br>-Ether oxygen of propranolol with hydroxyl group of Ser 203 (3.30 Ǻ).<br>-β-Hydroxyl group with hydroxyl group of Ser 204 (2.70 Ǻ), amino group of Ser 204 (2.86 Ǻ) and with amino group of Ser 203 (3.22 Ǻ).<br>-Terminal amino group with hydroxyl group of Ser 204 (2.85 Ǻ).<br>**Hydrophobic interactions:**<br>Naphthyl ring of propranolol and Phe 290.
**Falintolol** 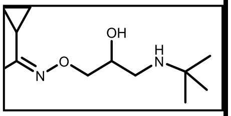 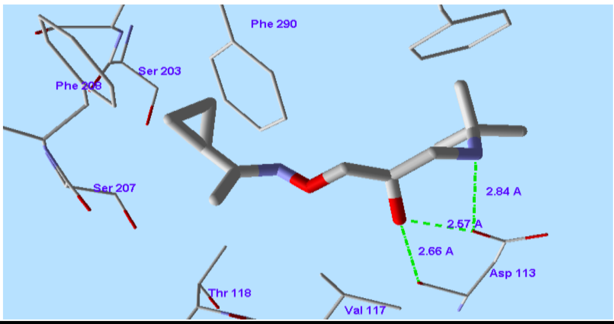	**Hydrogen bonding interactions:**<br>-β-Hydroxyl group of falintolol with carbonyl of free carboxylic group of Asp 113 (2.57 Ǻ) and with peptide carbonyl group of Asp 113 (2.66 Ǻ)<br>-Terminal amino group with carbonyl of free carboxylic group of Asp 113 (2.84 Ǻ)
**IPS 339** 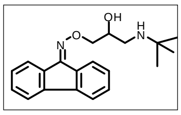 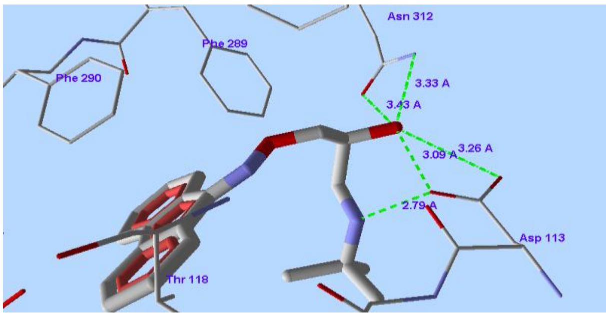	**Hydrogen bonding interactions:**<br>-β-Hydroxyl group in IPS 339 with carbonyl of free carboxylic group of Asp 113 (3.09 Ǻ), hydroxyl of free carboxylic group of Asp 113 (3.26 Ǻ), amino of amide group of Asn 312 (3.33 Ǻ) and with carbonyl of amide group of Asn 312 (3.43 Ǻ).<br>-Terminal amino group with carbonyl of free carboxylic group of Asp 113 (2.79 Ǻ).<br>**Ionic interaction:**<br>Terminal amino with carboxylate ion of Asp 113.<br>**Hydrophobic interactions:**<br>Aromatic ring with Phe 290 and Phe 289

Table S1. *Cont.*

<table>
<tr><th>Compound number/ structure/ docking snapshot</th><th>Forces involved in the ligand-receptor interaction</th></tr>
<tr><td>

4a

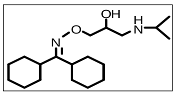

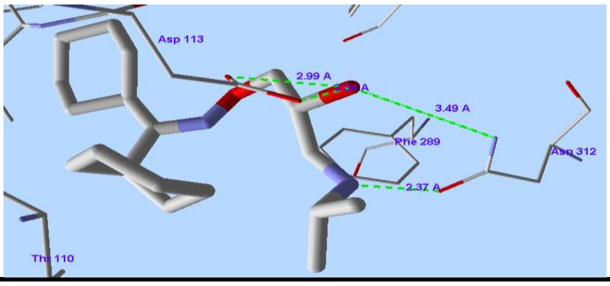

</td><td>

Hydrogen bonding interactions:

-β-Hydroxyl group in **83a** with carbonyl of free carboxylic group of Asp 113 (2.99 Å), hydroxyl of free carboxylic group of Asp 113 (3.34 Å), amino of amide group of Asn 312 (3.49 Å).

-Terminal amino group with peptide carbonyl group of Asn 312 (2.37 Å).

</td></tr>
<tr><td>

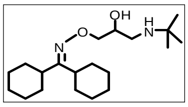

4b

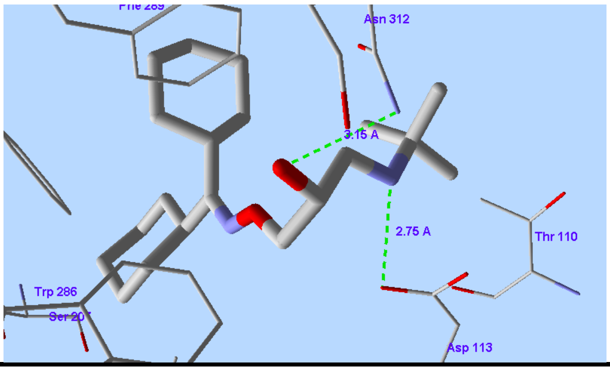

</td><td>

Hydrogen bonding interactions:

-β-Hydroxyl group in **83b** with amino of amide group of Asn 312 (3.15 Å).

-Terminal amino group with carbonyl of free carboxylic group of Asp 113 (2.75 Å).

Ionic interaction:

Terminal amino group with carboxylate ion of Asp 113.

</td></tr>
<tr><td>

4c

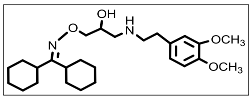

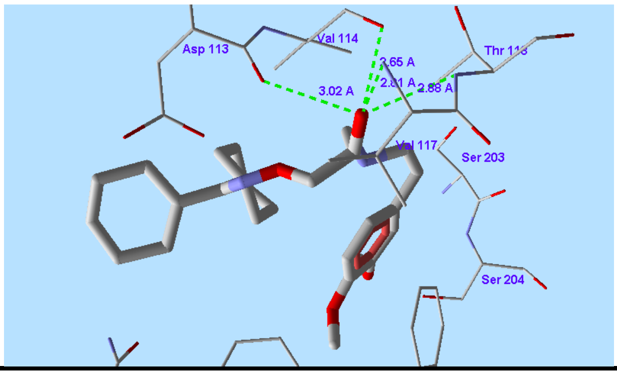

</td><td>

Hydrogen bonding interactions:

-β-Hydroxyl group in **83c** with amino of group of Thr 118 (2.88 Å), amino group of Val 117 (2.81 Å), carbonyl group Val 114 (2.65 Å) and with peptide carbonyl group of Asp 113 (3.02 Å).

</td></tr>
</table>

Table S1. *Cont.*

Compound number/ structure/ docking snapshot	Forces involved in the ligand-receptor interaction
**4d** 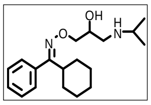 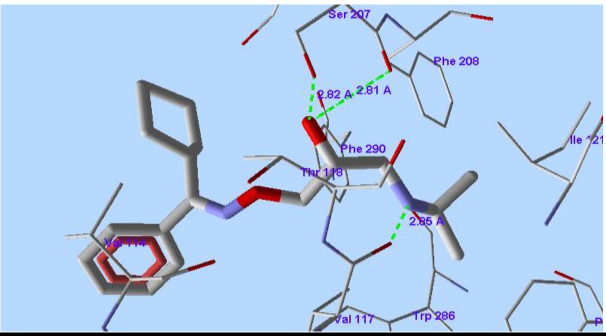	**Hydrogen bonding interactions:**<br>-β-Hydroxyl group in **84a** with hydroxyl group Ser 207 (2.82 Å) and with carbonyl of Ser 207 (2.81 Å).<br>-Terminal amino group with peptide carbonyl of Val 117 (2.85 Å).
**4e** 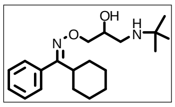 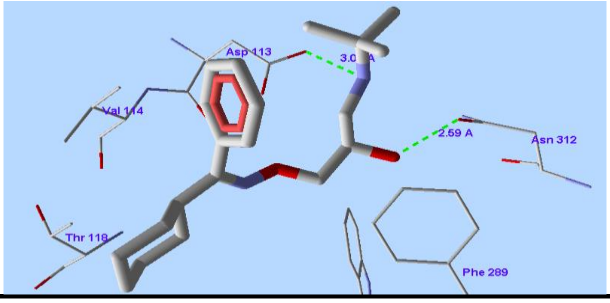	**Hydrogen bonding interactions:**<br>-β-Hydroxyl group in **84b** with amino of amide group of Asn 312 (2.59 Å).<br>-Terminal amino group with hydroxyl of free carboxylic group of Asp 113 (3.00 Å).<br>**Ionic interaction:**<br>Terminal amino group with carboxylate ion of Asp 113.
**4f** 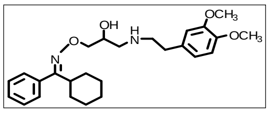 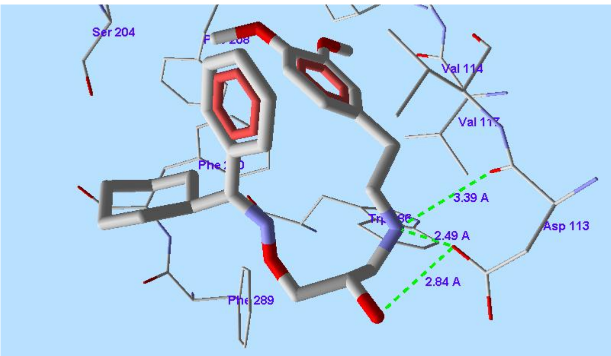	**Hydrogen bonding interactions:**<br>-β-Hydroxyl group in **84c** with carbonyl of free carboxyl group of Asp 113 (2.84 Å).<br>-Terminal amino group with peptide carbonyl group of Asp 113 (3.39 Å), carbonyl of free carboxyl group of Asp 113 (2.49 Å).<br>**Ionic interaction**:<br>Terminal amino group with carboxylate ion of Asp 113.

Table S1. *Cont.*

Compound number/ structure/ docking snapshot	Forces involved in the ligand-receptor interaction
**4g** 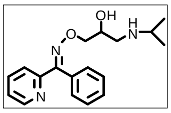 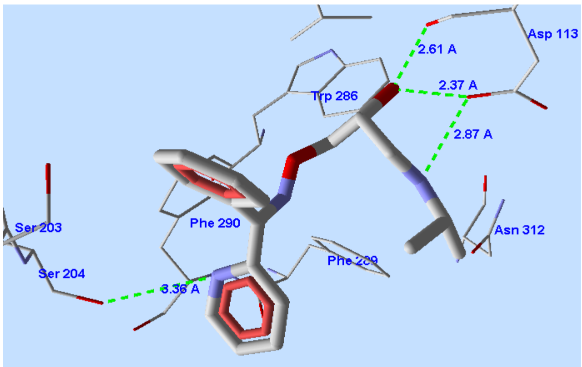	**Hydrogen bonding interactions:**<br>-Nitrogen in pyridine residue of **85a** with hydroxyl group of Ser 204 (3.36 Å).<br>-β-Hydroxyl group with carbonyl of free carboxyl group of Asp 113 (2.37 Å) and with peptide carbonyl group of Asp 113 (2.61 Å).<br>-Terminal amino group with carbonyl of free carboxyl group of Asp 113 (2.87 Å).<br>**Ionic interaction:**<br>Terminal amino group with carboxylate ion of Asp 113.<br>**Hydrophobic interactions:**<br>Pyridine ring with Phe 289 and Phe 290.
**4h** 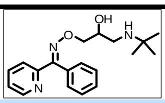 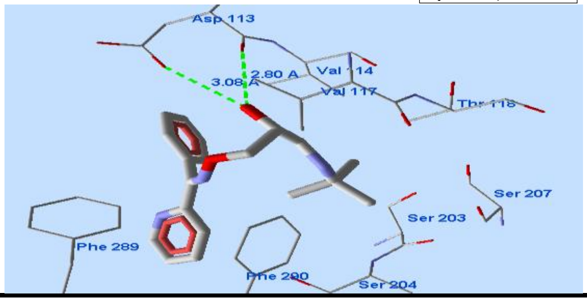	**Hydrogen bonding interactions:**<br>-β-Hydroxyl group in **85b** with carbonyl of free carboxyl group of Asp 113 (3.08 Å) and with peptide carbonyl group of Asp 113 (2.80 Å).<br>**Hydrophobic interactions:**<br>Pyridine ring and Phe 290 and Phe 289.
**4i** 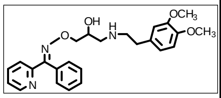 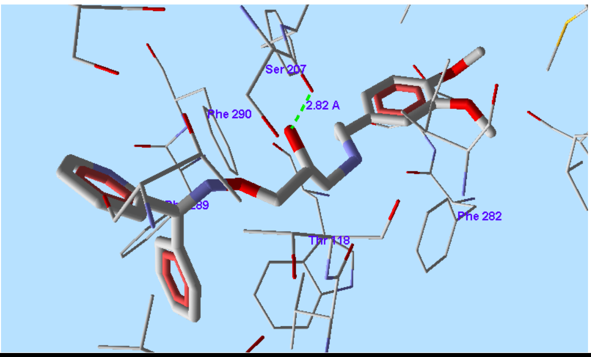	**Hydrogen bonding interactions:**<br>-β-Hydroxyl group in **85c** with carbonyl of Ser 207 (2.82 Å).<br>**Hydrophobic interactions:**<br>Phenyl ring of oxime with Phe 290 and Phe 289.

Table S1. *Cont.*

Compound number/ structure/ docking snapshot	Forces involved in the ligand-receptor interaction
**4k** 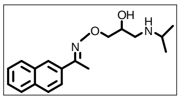 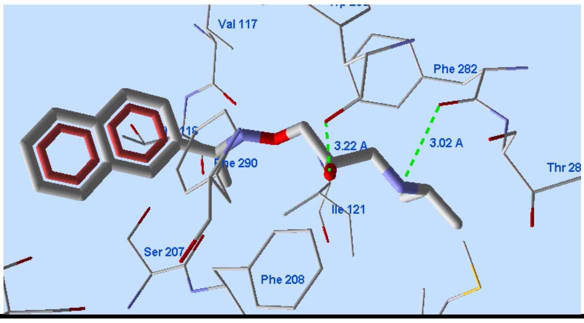	**Hydrogen bonding interactions:**<br>-β-Hydroxyl group in **86a** with carbonyl of Trp 286 (3.22 Ǻ).<br>-Terminal amino group with carbonyl group of Phe 282 (3.02 Ǻ).<br>**Hydrophobic interactions:**<br>Naphthyl ring with Phe 290
**4l** 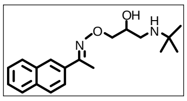 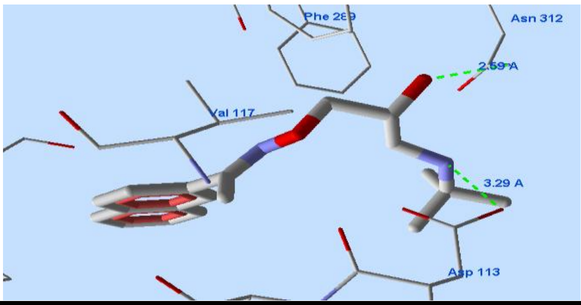	**Hydrogen bonding interactions:**<br>-β-Hydroxyl group in **86b** with amino of amide group of Asn 312 (2.59 Ǻ).<br>-Terminal amino group with hydroxyl of free carboxylic group of Asp 113 (3.29 Ǻ).<br>**Ionic interaction:**<br>Terminal amino group with carboxylate ion of Asp 113.
**4m** 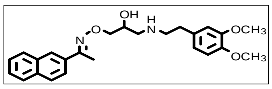 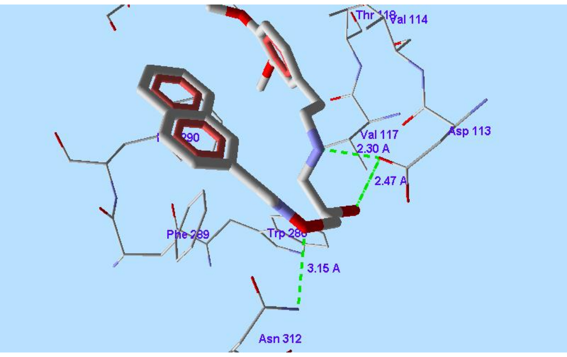	**Hydrogen bonding interactions:**<br>-Ether oxygen with of **86c** amino of amide group of Asn 312 (3.15 Ǻ).<br>-β-Hydroxyl group with carbonyl of free carboxylic group of Asp 113 (2.47 Ǻ).<br>-Terminal amino group with carbonyl of free carboxylic group of Asp 113 (2.30 Ǻ).<br>**Ionic interaction:**<br>Terminal amino group with carboxylate ion of Asp 113.<br>**Hydrophobic interactions:**<br>Naphthyl ring with Phe 290 and Phe 289.

Table S1. *Cont.*

Compound number/ structure/ docking snapshot	Forces involved in the ligand-receptor interaction
**4n** 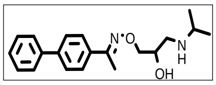 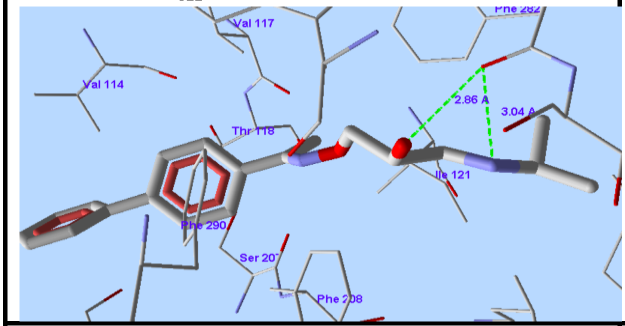	**Hydrogen bonding interactions:**<br>-β-Hydroxyl group in **87a** with carbonyl of Phe 282 (3.04 Ǻ).<br>-Terminal amino group with carbonyl of Phe 282 (2.86 Ǻ).
**4o** 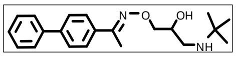 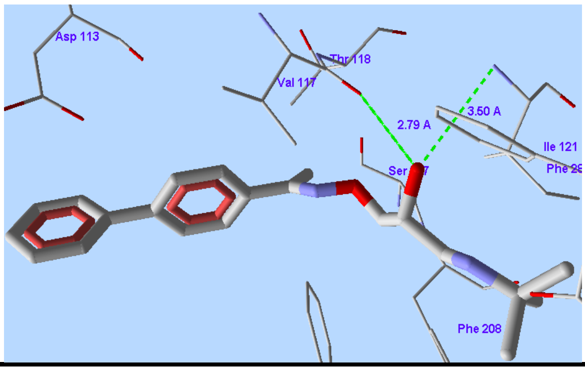	**Hydrogen bonding interactions:**<br>-β-Hydroxyl group in **87b** with carbonyl of Val 117 (2.79 Ǻ) and Ile 121 (3.50 Ǻ).
**4p** 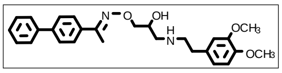 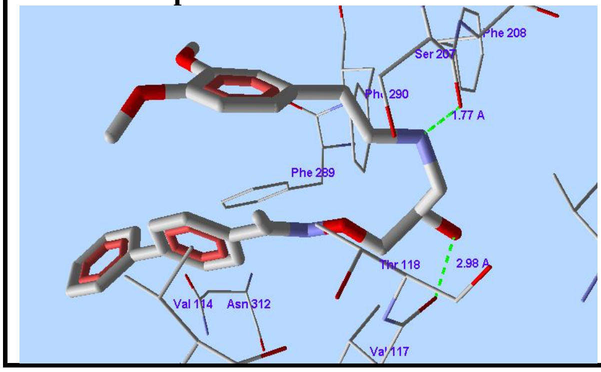	**Hydrogen bonding interactions:**<br>-β-Hydroxyl group in **87c** with carbonyl of Val 117 (2.98 Ǻ).<br>-Terminal amino group with carbonyl of Ser 207 (1.77 Ǻ).

Table S1. *Cont.*

Compound number/ structure/ docking snapshot	Forces involved in the ligand-receptor interaction
**4p** 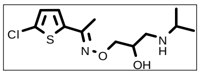 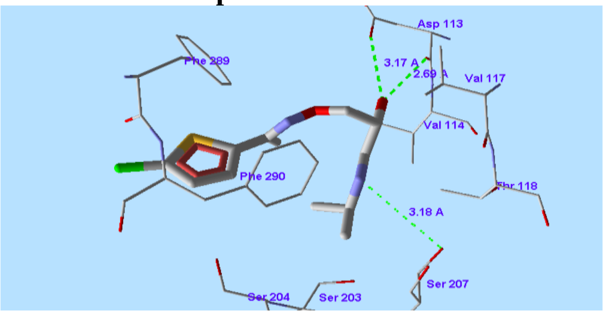	**Hydrogen bonding interactions:**<br>-β-Hydroxyl group in **88a** with peptide carbonyl group of Asp 113 (3.17 Å) and with carbonyl group of free carboxylic group of Asp 113 (2.69 Å).<br>-Terminal amino group with carbonyl group of Ser 207 (3.18 Å).<br>**Hydrophobic interactions:**<br>Thiophene ring with Phe 289 and Phe290.
**4q** 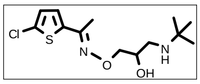 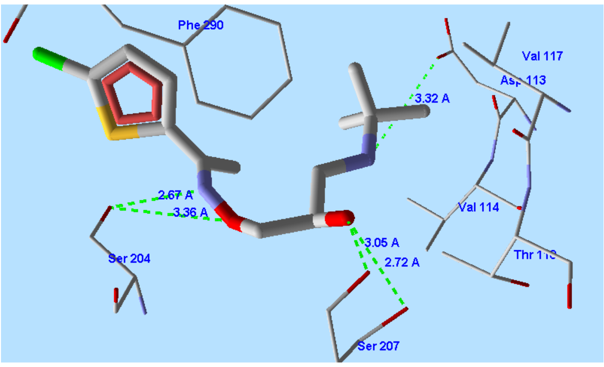	**Hydrogen bonding interactions:**<br>-Nitrogen of oxime in **88b** with hydroxyl group of Ser 204 (2.67 Å).<br>-Oxygen of oxime ether with hydroxyl group of Ser 204 (3.36 Å).<br>-β-Hydroxyl group with carbonyl of Ser 207 (3.05 Å) and with hydroxyl group of Ser 207 (2.72 Å).<br>-Terminal amino with carbonyl group of free carboxylic acid of Asp 113 (3.32 Å)<br>**Ionic interaction:**<br>Terminal amino group with carboxylate ion of Asp 113.<br>**Hydrophobic interactions:**<br>Thiophene ring with Phe 290
**4r** 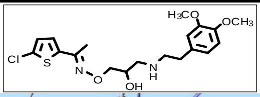 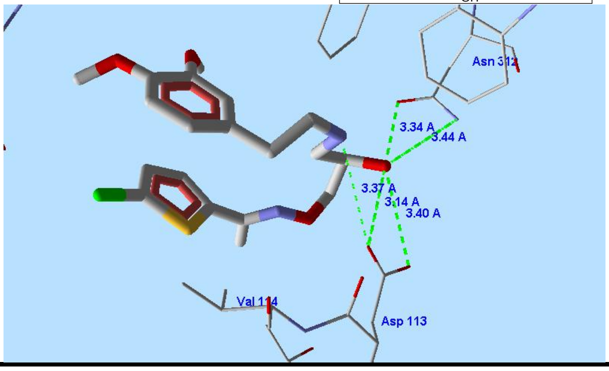	**Hydrogen bonding interactions:**<br>-β-Hydroxyl group in **88c** with carbonyl of amide group of Asn 312 (3.34 Å), amino of amide group of Asn 312 (3.44 Å), hydroxyl group of Asp 113 (3.40 Å) and with carbonyl of free carboxylic group of Asp 113 (3.14 Å).<br>-Terminal amino group with with carbonyl of free carboxylic group of Asp 113 (3.34 Å).

Table S1. *Cont.*

Compound number/ structure/ docking snapshot	Forces involved in the ligand-receptor interaction
**4s** 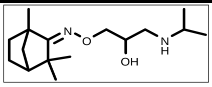 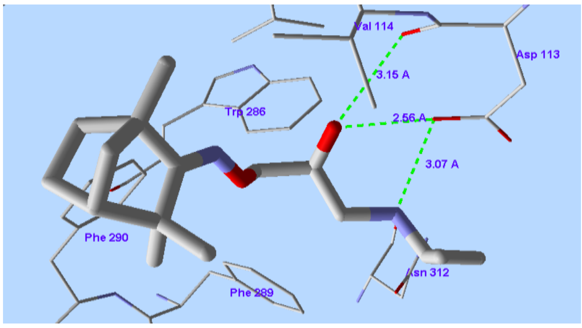	**Hydrogen bonding interactions:**<br>-β-Hydroxyl group in **89a** with carbonyl of free carboxylic group of Asp 113 (2.56 Ǻ) and with peptide carbonyl group of Asp 113 (3.15 Ǻ).<br>-Terminal amino group with carbonyl of free carboxylic group of Asp 113 (3.07 Ǻ).<br>**Ionic interaction:**<br>Terminal amino group with carboxylate ion of Asp 113.<br>**Hydrophobic interactions:**<br>bicyclic system with Phe 290.
**4t** 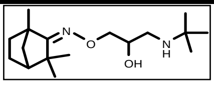 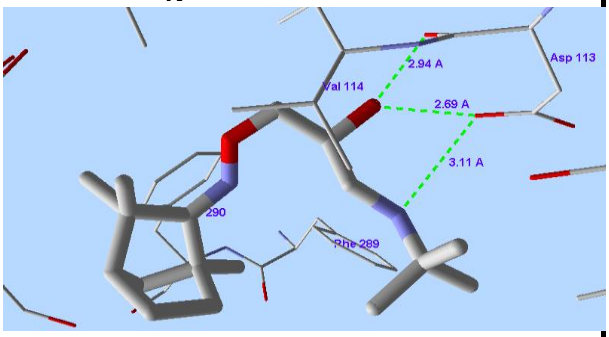	**Hydrogen bonding interactions:**<br>-β-Hydroxyl group in **89b** with carbonyl of free carboxylic group of Asp 113 (2.69 Ǻ) and with peptide carbonyl group of Asp 113 (2.94 Ǻ).<br>-Terminal amino group with carbonyl of free carboxylic group of Asp 113 (3.11 Ǻ).<br>**Ionic interaction:**<br>Terminal amino group with carboxylate ion of Asp 113.<br>**Hydrophobic interactions**<br>bicyclic system with Phe 290.
**4u** 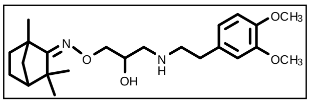 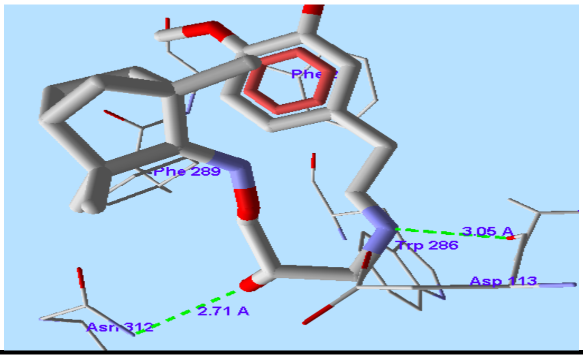	**Hydrogen bonding interactions:**<br>-β-Hydroxyl group of **89c** with amino of amide group of Asn 312 (2.71 Ǻ).<br>-Terminal amino group with peptide carbonyl group of Asp 113 (3.05 Ǻ).

Table S1. *Cont.*

Compound number/ structure/ docking snapshot	Forces involved in the ligand-receptor interaction
**4v** 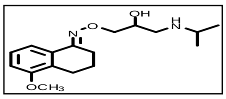 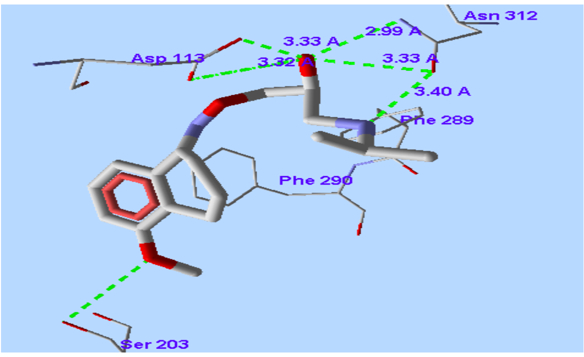	**Hydrogen bonding interactions:**<br>-5-methoxy group of tetralone in **90a** with hydroxyl group of Ser 203 (3.53 Å).<br>-β-Hydroxyl group with carbonyl of amide group of Asn 312 (3.33 Å), amino of amide group of Asn 312 (2.99 Å), carbonyl of free carboxylic group of Asp 113 (3.32 Å) and with hydroxyl of free carboxylic group of Asp 113 (3.33 Å).<br>-Terminal amino group with carbonyl of amide group of Asn 312 (3.40 Å).<br>**Hydrophobic interactions:**<br>Phenyl of tetralone residue with Phe 290.
**4w** 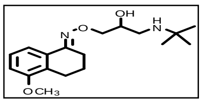 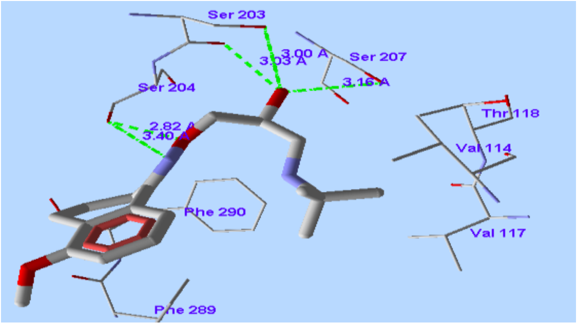	**Hydrogen bonding interactions:**<br>-Oxime Nitrogen of **90b** with hydroxyl group of Ser 204 (3.40 Å).<br>-Ether oxygen with hydroxyl group of Ser 204 (2.82 Å).<br>-β-Hydroxyl group with carbonyl of Ser 203 (3.03 Å), hydroxyl group of Ser 203 (3.00 Å) and with hydroxyl group of Ser 207 (3.16 Å).<br>**Hydrophobic interactions:**<br>Phenyl ring of tetralone residue and Phe 289.
**4x** 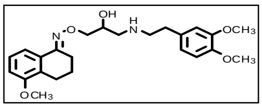 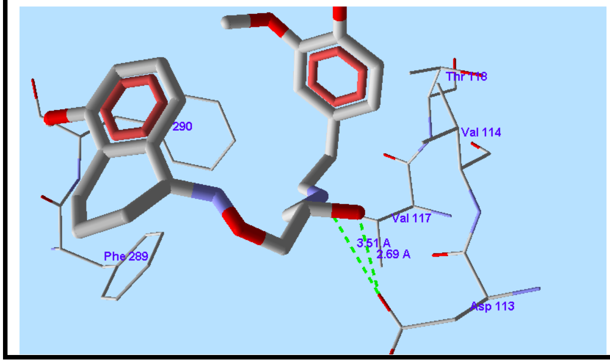	**Hydrogen bonding interactions:**<br>-β-Hydroxyl group in **90c** with carbonyl of free carboxylic group of Asp 113 (2.69 Å).<br>-Terminal amino group with carbonyl of free carboxylic group of Asp 113 (3.50 Å).<br>**Ionic interaction:**<br>Terminal amino group with carboxylate ion of Asp 113.<br>**Hydrophobic interactions:**<br>Phenyl ring of tetralone residue and Phe 289.

Table S1. *Cont.*

Compound number/ structure/ docking snapshot	Forces involved in the ligand-receptor interaction
**4y** 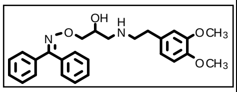 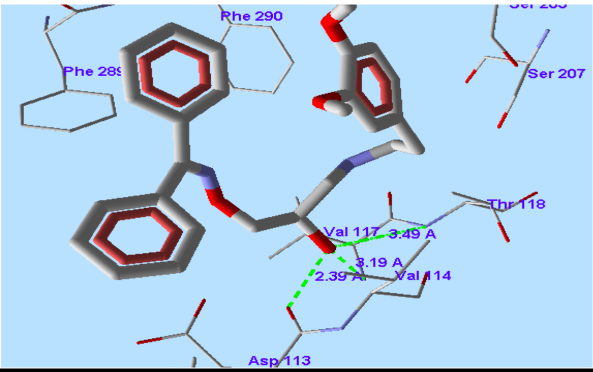	**Hydrogen bonding interactions:**<br>-β-Hydroxyl group in **91c** with peptide carbonyl group of Asp 113 (2.39 Å), amino of Val 117 (3.19Å) and with amino of Thr 118 (3.49 Å).<br>**Hydrophobic interactions:**<br>Phenyl of benzophenone residue with Phe 290 and Phe 289.
**4z** 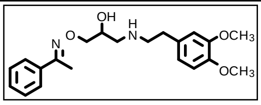 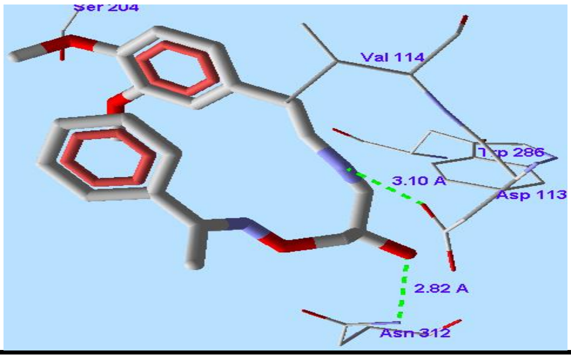	**Hydrogen bonding interactions:**<br>-β-Hydroxyl group in **92c** with amino of amide group of Asn 312 (2.82 Å).<br>-Terminal amino group with carbonyl of free carboxylic group of Asp 113 (3.10 Å).<br>**Ionic interaction:**<br>Terminal amino group with carboxylate ion of Asp 113
